# The impact of body mass index on health service utilization among pilgrims: a comprehensive analysis

**DOI:** 10.3389/fepid.2026.1769947

**Published:** 2026-08-10

**Authors:** Fahad Alamri, Reem Hasan, Areej Alshamrani, Lamis Al Abdulatif, Jumanah Alhazmi, Mariyyah Alburayh, Abrar Alkhashi, Hala Aljishi, Ahmed ElGanainy, Sami M. Alsaedi, Ayman Banjar

**Affiliations:** 1 Global Center for Mass Gathering Medicine (GCMGM), Riyadh, Saudi Arabia; 2 Public Health Authority, Riyadh, Saudi Arabia

**Keywords:** body mass index, Hajj, healthcare utilization, mass gatherings, obesity, underweight

## Abstract

**Background:**

Body Mass Index (BMI), a measure based on weight and height, is a key indicator for assessing health status and the risk of chronic diseases such as hypertension, diabetes, and cancer. Both ends of the BMI distribution have been associated with greater healthcare utilization, with prior evidence in general populations suggesting a non-linear (U-shaped) association in which underweight and obese individuals use more healthcare services than those with normal weight. This relationship is particularly important during mass gatherings like the Hajj pilgrimage, where overcrowding, extreme heat, and physical exertion can amplify health risks. This study aimed to investigate how variations in BMI influence clinic visits, hospital stays, and diagnoses among pilgrims during Hajj.

**Methods:**

A cross-sectional study was conducted among 4,000 pilgrims randomly selected from the official Hajj registry during the 2024 (1445 H) season. BMI was modelled as a categorical variable (<18.5, 18.5–24.9, 25–29.9, and ≥30 kg/m²), with the normal-weight category as the reference, allowing detection of non-linear (U-shaped) associations. Outcomes were clinic visit (yes/no), hospital admission (yes/no), and primary diagnosis, all collected using a standardized data collection form. Crude and adjusted odds ratios (cOR, aOR) with 95% confidence intervals and exact p-values were estimated using multiple logistic regression, adjusting simultaneously for age, gender, education, WHO region, marital status, employment, and smoking.

**Results:**

Underweight (BMI <18.5) and obese (BMI ≥30) pilgrims reported higher rates of clinic visits (9% and 10%, respectively) and higher rates of hospital admission than normal-weight and overweight pilgrims, confirming a U-shaped association between BMI and healthcare utilization. The most frequent diagnoses were heat-related illnesses (75% of admissions among underweight, 76% among obese), followed by chronic-disease follow-up, particularly at BMI extremes. In the adjusted analyses, WHO region and employment status remained significantly associated with clinic visits, with lower odds among pilgrims from South-East Asia and higher odds among unemployed individuals with BMI <18.5.

**Conclusion:**

Both low and high BMI are linked to greater healthcare utilization during Hajj. Implementing targeted risk assessments, pre-travel health education, and culturally adapted healthcare services for BMI extremes may reduce acute healthcare demand during mass gatherings.

## 1 Introduction

The body mass index, or BMI, is essentially a weight-and-height-based indicator of body fat (1).

To estimate a person’s health status, BMI is calculated by using height and weight values based on mathematical operations (2). According to the World Health Organization (WHO), it defines overweight as having a BMI of 25 or higher, obesity as having a BMI of 30 or higher, and underweight as having a BMI of less than 18.5 (1).

Given these classifications, the health risks associated with overweight and obesity are increasingly well documented and understood (3, 13). Therefore, these classifications can be used to assess conditions such as hypertension, diabetes, cancer, hypercholesterolemia, and other chronic diseases, making BMI a critical public health indicator (2). Individual with high BMI are at greater risk for developing non-communicable diseases (1). Hence, increased healthcare utilization may be needed to manage these conditions.

This correlation is supported by previous observational studies that have demonstrated a robust positive relationship between elevated BMI and increased healthcare utilization. For instance, a Canadian study of obese and morbidly obese individuals found that they were more likely to report having serious chronic conditions. Notably, only the morbidly obese (BMI >35 kg/m^2^) group showed a significantly higher number of visits to a general practitioner over a five-year period compared to the normal weight group (4).

Similarly, a cross-sectional, nationally representative study in Brazil indicate that Individuals with obesity (both male and female) used nearly twice as many healthcare services as those with under/normal weight (5). The study found that obese men with hypertension faced a higher risk of hospital admissions than their under/normal weight counterparts. Additionally, women with obesity and diabetes had more consultations with specialists compared to those with underweight and normal-weight. Also, retrospective research in family medicine practices showed that BMI ≥ 35 independently increased odds of being a “frequent visitor” (top quartile of visit rates), even when controlling for comorbidities and demographics (6). Lifetime utilization estimates show those with BMI ≥ 35 make over 100% more primary-care contacts than normal-weight peers  (7).

The relationship between BMI and healthcare use becomes even more critical in mass gathering events such as the Hajj pilgrimage. Hajj, one of the five pillars of Islam, annually draws over 1.8 million pilgrims from around the world to Makkah, Saudi Arabia (8). Such an event represents a significant public health challenge due to Overcrowding, high temperatures, physical exertion, limited sleep, the presence of many elderly participants and those with pre-existing health conditions (9). In a cross-sectional study among French pilgrims, nearly 75% were either overweight or obese, and obesity was more prevalent in individuals aged over 60 years  (10).

Even though there is plenty of evidence from general populations, there are almost no studies that have examined the connection between BMI and clinic visits during mass gatherings, such as Hajj. It is known that Hajj pilgrims are more likely to be overweight or obese, have hypertension, and suffer from diabetes. Crowded environments, extreme climate conditions, lack of adequate sleep, as well as physical strain intensify the health risks for individuals with high BMI.

It is important to note that the prior evidence cited above derives from heterogeneous health-system contexts. The Canadian estimates (Twells et al.) come from a universal-coverage primary-care system; the Brazilian data (Rimes-Dias et al.) from the National Health Survey; the US figures (Baker et al.) from private family-medicine practices; and the lifetime-utilization estimate (Edwards et al.) from a long-term Norwegian cohort under a universal-coverage system. The magnitude of the BMI–utilization association is therefore expected to vary with the structure of the underlying health system, and direct quantitative comparison with a short-duration religious mass-gathering setting in Saudi Arabia must be interpreted with caution. Furthermore, while the evidence above documents elevated utilization at higher BMI, several studies in general populations also report increased healthcare use among underweight individuals, producing a non-linear (U-shaped) association between BMI and healthcare utilization that is biologically plausible under the specific physical and environmental stressors of Hajj (7, 11, 12).

Importantly, prior evidence in general populations suggests that the relationship between BMI and healthcare utilization may not be strictly linear: both underweight and obese individuals tend to use more services than normal-weight peers, producing a U-shaped association. Under the physiological and environmental stressors specific to mass gathering like Hajj — extreme ambient temperatures, sustained physical exertion, overcrowding, sleep deprivation, and infectious-disease transmission risk — such a non-linear pattern is biologically plausible. This study therefore aims to fill the existing evidence gap by investigating how BMI influences clinic visits, hospital admission, and primary diagnoses among pilgrims during the Hajj pilgrimage in Makkah. We hypothesized, in an exploratory and non-directional manner, that pilgrims with BMI values outside the normal range — both underweight and obese — would exhibit a higher frequency of clinic visits and higher rates of hospital admission during Hajj compared with normal-weight pilgrims.

## 2 Method

### 2.1 Study design

This cross-sectional study included a simple random sample of 4,000 pilgrims participating in the 2024 Hajj season (1445 AH) in Makkah, Saudi Arabia. Participants were selected from the official Hajj registry provided by the Saudi Ministry of Hajj using a computer-generated random-number sequence. Data were collected from health records and foreign medical teams using a standardized data collection form adapted to the study objectives. Data collection was conducted between May 9 and July 21, 2024, to capture the full pilgrim journey, including the pre-Hajj arrival period, the core 5-day Hajj ritual period, and the immediate post-Hajj period before departure from Saudi Arabia. Healthcare utilization was therefore assessed throughout participants’ stay in Makkah from arrival to departure. Ethical principles outlined in the Declaration of Helsinki were followed, and ethical approval was obtained from King Fahad Medical City (IRB No. 25-661E).

### 2.2 Sample size

The minimum required sample size was determined *a priori* using the **Raosoft®** sample size calculator, assuming a population of 1,673,230 pilgrims, a 95% confidence level, a 50% response distribution, and a 1.5% margin of error.‏ This yielded a target sample size of 4,258 participants.‏

To account for anticipated non-response and eligibility exclusions, a larger sample of 8,159 pilgrims was approached through the official Hajj registry. Of these, 4,159 individuals were not included in the final analysis due or incomplete form. These categories were recorded collectively in the records and therefore could not be reported separately.‏ The final analytic sample comprised 4,000 completed, corresponding to a realised margin of error of approximately 1.55% at the 95% confidence level, only 0.05 percentage points higher than the planned precision. In addition, a *post hoc* power analysis based on the observed association between BMI and clinic visits demonstrated that the achieved sample size provided more than 80% statistical power at a significance level of *α* = 0.05

### 2.3 Data collection

Data was collected via a health records by trained research assistants through a standardized data collection form. All participants data were anonymous and did not include personally identifiable information. The data collection form contained three sections: (i) sociodemographic items (gender, age, WHO region, education, marital status, employment status, smoking status); (ii) anthropometric items self-reported height and weight were verified with the assistance of foreign medical teams; and (iii) healthcare-utilization items (number of clinic visits, hospital admission, length of stay, and primary diagnosis). The instrument was developed in English, informed by prior literature on BMI and healthcare utilization, and adapted to the Hajj context. It was pilot-tested in a small subset of pilgrims prior to full deployment, with minor wording adjustments made on the basis of pilot feedback. Research assistants were native or fluent speakers of Arabic, English, and additional pilgrim languages, and live interpretation was provided where needed for languages not covered by the field team. All research assistants completed a standardized one-day training session on health record data collection procedures and data confidentiality prior to data collection to ensure standardized data collection.

### 2.4 Anthropometric measurement

Height and weight were obtained by self-report documented in health records received from foreign medical teams, because the field conditions during Hajj (large crowd density, time constraints, mobility of participants, and cultural considerations around gender-segregated measurement) did not permit standardized direct measurement of 4,000 individuals. To minimize misclassification, the recorded self-reported (within the past 6 months) when available, was used, and the documented height and weight values were verified with the assistance of foreign medical teams.

### 2.5 Statistical analysis

Descriptive statistics were used to summarize the demographic and clinical characteristics of the participants. Categorical data were presented as counts and percentages, while continuous data were reported as means with standard deviations. The t-test was applied for continuous variables To examine factors associated with the frequency of clinic visits during Hajj, multiple logistic regression analyses were conducted, yielding odds ratios (OR), adjusted odds ratios (aOR), and 95% confidence intervals (CI). A significance threshold of *p* < 0.05 was set. Statistical analyses were performed using MATLAB (R2023b version 23.2). BMI was stratified into four categories (<18.5, 18.5–24.9, 25–29.9, and ≥30 kg/m²), and separate multivariate logistic regression models were conducted for each BMI category. The primary binary outcome was clinic visit (yes/no). The following covariates were entered simultaneously into the multivariate logistic regression models: age (continuous), gender, education level, nationality, marital status, employment status, and smoking status. These variables were selected *a priori* based on biological and epidemiological plausibility and prior literature on healthcare-utilization determinants. No stepwise variable-selection procedure was applied. For the primary binary outcome, each participant was counted only once regardless of the number of clinic visits made.

## 3 Result

### 3.1 Participant characteristics

As shown in Table 1 the study involved 4,000 patients, with an equal gender distribution of 50% male and 50% female, and an average age of 55 years (±13). In terms of education, 5% were illiterate, 55% had a primary education, 39% completed intermediate or secondary education, and only 1% were university graduates. The participants were primarily from the Eastern Mediterranean (29%), South-East Asia (30%), and the African Region (16%), with a small representation from the Americas (0.05%) and Europe (10%). Regarding marital status, 48% were married, while 52% were not. Employment status revealed that 58% were employed and 42% were unemployed. Smoking rates indicated that 11% of participants were smokers, while 89% were non-smokers. Finally, only 9% reported taking medical consultations, highlighting a significant portion (91%) who did not seek care.

**Table 1:** Pilgrims’ socio-demographics and healthcare visits (n = 4,000 patients).

	Reported without Chronic Disease	
Characteristic	N	%
Age (range 18 – 100), years	55 ± 13	
Gender		
Female	2,000	50
Male	2,000	50
Education level		
Illiterate	193	5
Primary	2,180	55
Intermediate or Secondary	1,576	39
University	51	1
WHO Regions	***N*** **=** **3984**^a^	
Eastern Mediterranean	1,146	29
African	634	16
South-East Asia	1,181	30
Western Pacific	626	16
Region of the American	2	0.05
European	395	10
Marital Status		
Married	1,907	48
Not Married	2,093	52
Employment Status		
Yes	2,306	58
No	1,694	42
Smoking Status		
Yes	425	11
No	3,575	89
Healthcare Visits		
Yes	351	9
No	3,649	91

a Sixteen participants (*n* = 16) were excluded from the WHO Region row of Table 1 because their region of origin was missing or could not be assigned; the denominator for this row is therefore *N* = 3,984. All other variables retain the full *n* = 4,000.

### 3.2 Relationship between body mass Index (BMI) and number of healthcare visits

**Figure 1:**
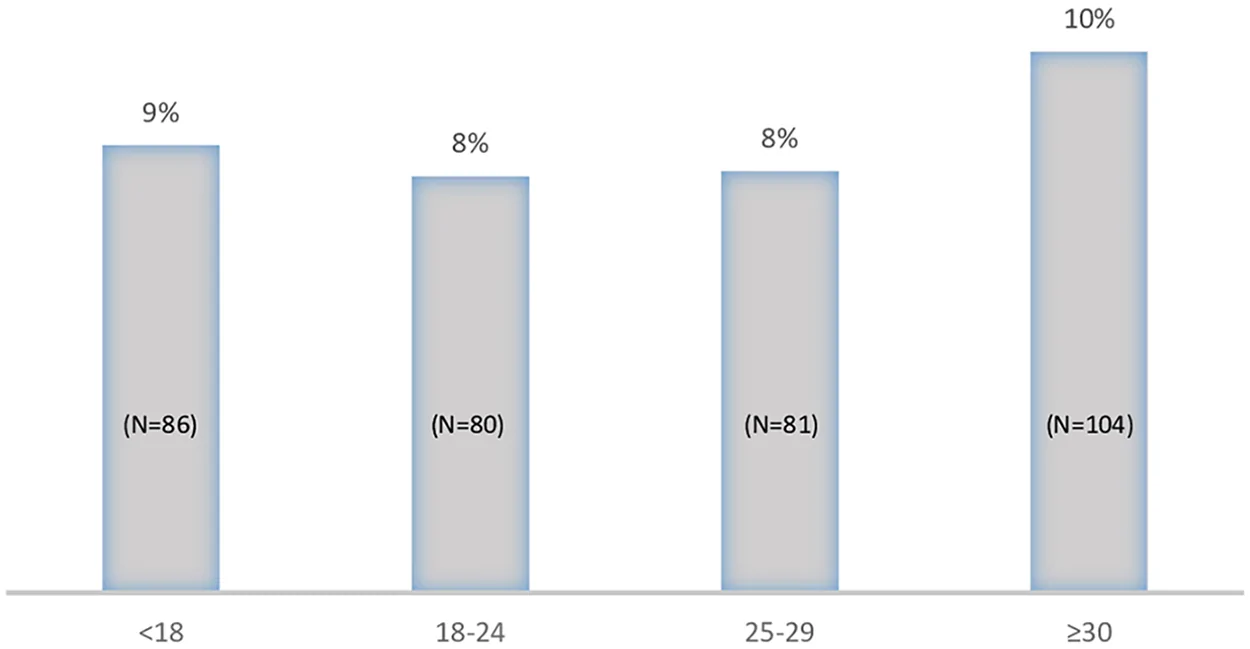
Distribution of healthcare visits (*N* = 352) by body mass Index (BMI) categories.

Figure 1 illustrates the relationship between Body Mass Index (BMI) and clinic visits. The proportion of pilgrims reporting at least one clinic visit was 9% in the underweight category (BMI <18.5), 8% in the normal-weight category (BMI 18.5–24.9), 8% in the overweight category (BMI 25–29.9), and 10% in the obese category (BMI ≥30). The pattern is consistent with the U-shaped association between BMI and healthcare utilization introduced in the Methods: both underweight and obese pilgrims showed higher clinic-visit rates than the normal-weight reference, while overweight pilgrims showed the same rate as normal-weight pilgrims.

### 3.3 The relationship between body mass Index (BMI) and hospital admission

The analysis presented in Table 2 demonstrates variations in hospitalization patterns across Body Mass Index (BMI) categories. Heat-related illnesses showed the highest frequency of hospital admissions in all BMI groups, accounting for 52% of admissions among underweight patients (BMI ≤ 18.5), 44% among individuals with a BMI of 18.5–24.9, 43% among those with a BMI of 25–29.9, and 55% among patients with a BMI ≥ 30. Chronic diseases were also commonly associated with hospitalization, representing 9% of admissions in both the underweight and overweight (BMI 25–29.9) groups, 5% in the normal BMI group, and 8% in the obese group. Respiratory diseases were most frequently observed among admitted patients with a BMI of 18.5–24.9, accounting for 8% of admissions in this category. Cardiovascular diseases were relatively uncommon across all BMI groups, with only 3% of admitted patients in the BMI 18.5–24.9 category and 1% in the BMI 25–29.9 category affected. Overall, these findings suggest that heat-related illnesses were the predominant cause of hospitalization regardless of BMI classification, while chronic and respiratory diseases showed moderate variation between BMI groups.

**Table 2:** Prevalence of diagnoses by body mass Index and hospital admission Status.

	≤18.5		18.5–24.9		25–29.9		≥30	
Body Mass Index (BMI)	Admitted	No	Admitted	No	Admitted	No	Admitted	No
*Hospital Admission Status*	*N (%)*	*N (%)*	*N (%)*	*N (%)*	*N (%)*	*N (%)*	*N (%)*	*N (%)*
Participants per group (n)	*n* = 86	*n* = 914	*n* = 80	*n* = 920	*n* = 81	*n* = 919	*n* = 104	*n* = 896
Cardiovascular Disease	0 (0%)	0 (0%)	2 (3%)	0 (0%)	1 (1%)	0 (0%)	0 (0%)	0 (0%)
Chronic Disease	8 (9%)	13 (1%)	4 (5%)	9 (1%)	7 (9%)	8 (1%)	8 (8%)	17 (2%)
Dermatological Disease	2 (2%)	0 (0%)	4 (5%)	0 (0%)	4 (5%)	0 (0%)	2 (2%)	0 (0%)
Heat-related Illnesses	45 (52%)	4 (0%)	35 (44%)	3 (0%)	35 (43%)	6 (1%)	57 (55%)	2 (0%)
Infectious Disease	0 (0%)	0 (0%)	3 (4%)	1 (0%)	2 (2%)	0 (0%)	4 (4%)	0 (0%)
Injuries	1 (1%)	0 (0%)	1 (1%)	0 (0%)	0 (0%)	0 (0%)	0 (0%)	0 (0%)
Neurological diseases	1 (1%)	0 (0%)	0 (0%)	0 (0%)	3 (4%)	0 (0%)	1 (1%)	0 (0%)
Obstetrics/Gynecological disease	1 (1%)	0 (0%)	2 (3%)	0 (0%)	1 (1%)	0 (0%)	1 (1%)	0 (0%)
Respiratory disease	1 (1%)	9 (1%)	6 (8%)	9 (1%)	2 (2%)	13 (1%)	2 (2%)	10 (1%)
Visual Disorders	1 (1%)	0 (0%)	0 (0%)	0 (0%)	0 (0%)	0 (0%)	0 (0%)	0 (0%)

### 3.4 Statistical analysis of contributing factors

The univariate analysis in Table 3 presents both the crude (cOR) and adjusted (aOR) odds ratios with 95% confidence intervals and exact p-values for each covariate, modelled separately within each BMI category. The dependent variable in this analysis is clinic visit (yes/no). In the adjusted (multivariate) analysis, pilgrims from the South-East Asia region showed significantly lower odds of clinic visits across all BMI categories (all *P* < 0.01). Conversely, pilgrims from the European region exhibited an adjusted odds ratio of 2.036 (*P* = 0.0260) within the underweight category (BMI < 18.5), consistent with a higher likelihood of clinic visits in this subgroup. Employment status was significantly associated with clinic visits within the underweight category: unemployed pilgrims had an aOR of 0.087 (*P* < 0.0001). These findings highlight the joint role of WHO region and employment status in shaping clinic-visit patterns across BMI groups.

**Table 3:** Univariate logistic regression of factors associated with clinic visit across (BMI) categories.

	Univariate Analysis			
	Unadjusted OR - (95%CI) P-value			
Factors	*BMI <18*	*BMI 18–24*	*BMI 25–29*	*BMI *≥* 30*
Age (range 18–100), years	0.99 (0.99–1) *P* = 0.19717^a^	1 (0.99–1) *P* = 0.25165^a^	1 (0.99–1) *P* = 0.287126^a^	0.9 (0.99–1) *P* = 0.60147^a^
Gender				
Female	0.654 (0.42–1.03) *P* = 0.0650^a^	0.84 (0.53–1.33) *P* = 0.4673^a^	0.89 (0.57–1.41) *P* = 0.6359^a^	0.9 (0.6–1.36) *P* = 0.6260^a^
Male	**Ref**	**Ref**	**Ref**	**Ref**
Education level				
Illiterate	0.49 (0.11–2.1) *P* = 0.329^a^	2.04 (0.91–4.61) *P* = 0.0843^a^	0.25 (0.03–1.83) *P* = 0.1711^a^	1.6 (0.68–3.79) *P* = 0.2742^a^
Primary	**Ref**	**Ref**	**Ref**	**Ref**
Intermediate or Secondary	1.16 (0.74–1.83) *P* = 0.5153^a^	1 (0.615–1.69) *P* = 0.9936^a^	0.75 (0.46–1.22) *P* = 0.2458^a^	0.76 (0.49–1.17) *P* = 0.2211^a^
University	0.61 (0.07–4.65) *P* = 0.6314^a^	0.69 (0.039–12.3) *P* = 0.8072^a^	1.1 (0.14–8.82) *P* = 0.9315^a^	0.6 (0.08–4.68) *P* = 0.6284^a^
WHO Regions				
Eastern Mediterranean	**Ref**	**Ref**	**Ref**	**Ref**
African	0.723 (0.38–1.36) *P* = 0.3123^a^	0.66 (0.32–1.35) *P* = 0.2520^a^	0.88 (0.44–1.75) *P* = 0.7115^a^	0.79 (0.45–1.37) *P* = 0.4059^a^
South-East Asia	0.18 (0.084–0.41) *P* < 0.0001^a^	0.36 (0.18–0.71) *P* = 0.0033^a^	0.33 (0.16–0.69) *P* = 0.0032^a^	0.2 (0.1–0.41) *P* < 0.0001^a^
Western Pacific	0.53 (0.25–1.1) *P* = 0.0900^a^	0.66 (0.33–1.36) *P* = 0.261^a^	0.76 (0.39–1.46) *P* = 0.4094^a^	0.9 (0.18–0.83) *P* = 0.0140^a^
European	2.036 (1.08–3.81) *P* = 0.0260^a^	1.1 (0.56–2.17) *P* = 0.7831^a^	1.5 (0.756–2.99) *P* = 0.2445^a^	0.92 (0.48–1.74) *P* = 0.7971^a^
Marital Status				
Married	0.844 (0.54–1.32) *P* = 0.455^a^	0.93 (0.59–1.46) *P* = 0.7512^a^	1 (0.65–1.61) *P* = 0.9230^a^	0.78 (0.52–1.18) *P* = 0.2340^a^
Not Married	**Ref**	**Ref**	**Ref**	**Ref**
Employment Status				
Yes	**Ref**	**Ref**	**Ref**	**Ref**
No	0.087 (0.04–0.21) *P* < 0.0001^a^	1 (0.64–1.62) *P* = 0.9247^a^	1 (0.72–1.81) *P* = 0.5827^a^	0.78 (0.52–1.17) *P* = 0.2382^a^
Smoking Status				
Yes	0.74 (0.35–1.57) *P* = 0.4380^a^	0.69 (0.29–1.64) *P* = 0.4079^a^	1.4 (0.71–2.73) *P* = 0.33^a^	0.95 (0.48–1.89) *P* = 0.8902^a^
No	**Ref**	**Ref**	**Ref**	**Ref**

a Statistically significant (*P* < 0·05).

**Figure 2:**
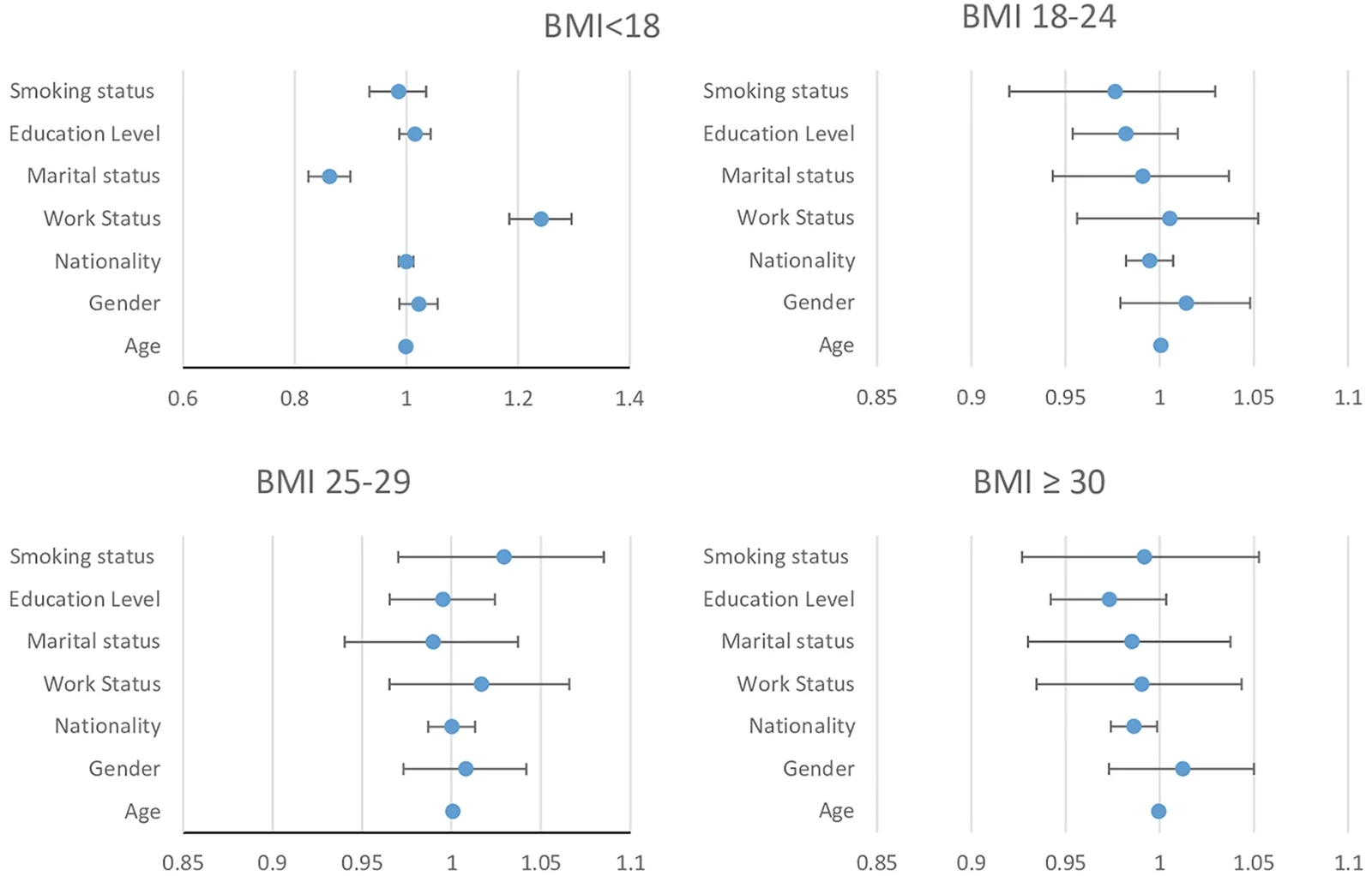
Forest plot of adjusted odds ratios (aOR) with 95% CI and p-values for factors associated with clinic visits across BMI categories, adjusted for covariates in multivariable logistic regression.

Table 4 and Figure 2 highlight the multivariable logistic regression findings for factors associated with clinic visits across BMI categories. The forest plot in Figure 2 was consistent with the results presented in Table 4. Among pilgrims with BMI <18.5, work status was significantly associated with increased odds of clinic visits, while marital status showed a significant inverse association. Among pilgrims with BMI ≥30, nationality remained significantly associated with clinic visits. No significant associations were observed for age, gender, education level, or smoking status across most BMI categories.

**Table 4:** Multinomial logistic regression of factors associated with BMI categories (aOR, 95% CI, p-values).

Variable	BMI <18.5 aOR (95% CI)	p-value	BMI 18.5–24.9 aOR (95% CI)	p-value	BMI 25–29.9 aOR (95% CI)	p-value	BMI ≥30 aOR (95% CI)	p-value
Age	0.999 (0.998–1.000)	0.121	1.001 (0.999–1.002)	0.265	1.001 (0.999–1.002)	0.262	1.000 (0.998–1.001)	0.543
Gender	1.022 (0.989–1.057)	0.197	1.014 (0.980–1.049)	0.415	1.008 (0.974–1.043)	0.645	1.012 (0.974–1.051)	0.530
Nationality	1.000 (0.987–1.013)	0.942	0.995 (0.982–1.007)	0.414	1.000 (0.987–1.013)	0.978	0.986 (0.974–0.999)	0.031^a^
Work Status	1.241 (1.187–1.298)	<0.001^a^	1.005 (0.958–1.055)	0.825	1.017 (0.968–1.068)	0.507	0.991 (0.938–1.046)	0.733
Marital Status	0.863 (0.826–0.901)	<0.001^a^	0.991 (0.945–1.039)	0.711	0.990 (0.943–1.040)	0.683	0.985 (0.933–1.040)	0.593
Education Level	1.016 (0.988–1.044)	0.269	0.982 (0.955–1.010)	0.212	0.995 (0.966–1.025)	0.753	0.973 (0.943–1.004)	0.092
Smoking Status	0.986 (0.936–1.038)	0.588	0.976 (0.923–1.033)	0.402	1.029 (0.973–1.088)	0.310	0.992 (0.931–1.057)	0.799

a Statistically significant (*P* < 0·05).

.

## 4 Discussion

This study examined the impact of Body Mass Index (BMI) on healthcare utilization during Hajj, a distinct mass gathering event characterized by physical exertion, heat exposure, and participation from diverse international backgrounds. According to the findings, BMI is associated with the frequency of clinic visits, hospital admission status, and primary diagnosis pattern. While our original expectation, framed as an exploratory and non-directional hypothesis, was that pilgrims at either extreme of the BMI distribution would exhibit greater healthcare utilization than normal-weight pilgrims, the data revealed a clear U-shaped association in which both underweight (BMI <18.5) and obese (BMI ≥30) groups showed elevated clinic-visit and admission rates compared to the normal-weight reference. This pattern is biologically coherent. Among underweight pilgrims, sarcopenia and reduced lean tissue mass, micronutrient depletion, reduced cardiopulmonary reserve, compromised immune competence, and impaired sweat-mediated thermoregulation each plausibly contribute to greater vulnerability under the physical and environmental demands of Hajj (13–15). Among obese pilgrims, impaired heat dissipation due to increased subcutaneous adipose insulation and a lower surface-area-to-mass ratio, elevated metabolic heat production during sustained physical exertion (long walking distances and ritual rounds), and a higher background burden of chronic comorbidities (hypertension, type 2 diabetes, cardiovascular disease, osteoarthritis) similarly elevate risk.

Firstly, both underweight (BMI <18.5) and obese (BMI ≥30) individuals exhibited higher rates of clinic visits compared to those with normal or overweight BMI, suggesting a U-shaped association between BMI and healthcare utilization. These **findings** are consistent with previous research in general populations, which demonstrates that both extremes of BMI are linked to increased use of health services and prolonged hospital stays (7, 16). For example, Edwards et al. reported that obese individuals, especially those with a BMI ≥35, had up to 102% higher lifetime healthcare utilization than their normal-weight peers (7). Similarly, underweight individuals with reduced lean tissue have been found to experience prolonged hospitalization due to limited physiological reserves (16). It should be noted, however, that the 102% lifetime-utilization estimate from Edwards et al. derives from a long-term Norwegian cohort under a universal-coverage health system, with a demographic structure, climatic exposure, and time horizon that differ substantially from a short-duration religious mass gathering in Saudi Arabia. This figure is therefore cited as illustrative of the magnitude of obesity-related lifetime utilization rather than as a directly comparable benchmark for our Hajj setting.

Secondly, the data suggest that the BMI ≥30 group was the most likely to be admitted to hospital. This is supported by hospital-based studies showing that obesity increases the risk of complications and prolongs recovery, leading to extended length of stay (17). A systematic review further demonstrated that obesity not only drives hospital utilization but also contributes to higher healthcare costs, with obese individuals incurring nearly 30% higher medical expenditures than their normal-weight peers (17)

Thirdly, common diagnoses across all BMI groups included upper respiratory infections and follow-ups for chronic conditions, with chronic disease management particularly dominant in the underweight and obese categories. Additional evidence suggests that obesity is linked to greater comorbidity burdens, such as hypertension, diabetes, and respiratory disease (3), while underweight status may reflect frailty or increased vulnerability to infections and chronic illnesses (18, 19). One study cited in a systematic review showed that respiratory tract infections were the leading cause of hospitalization in Saudi hospitals during the Hajj, with rates as high as 57% (20). The overlap between acute respiratory infections and chronic disease care highlights the dual challenge of managing communicable and non-communicable conditions in mass gatherings. Although data that explored the link between BMI and heat-related illness specifically during Hajj are limited, existing evidence highlights obesity as a known risk factor. Our findings show that HRI rates were similarly high among underweight (75%) and obese (76%) pilgrims, suggesting comparable vulnerability at both BMI extremes. Heat stress and BMI extremes. The near-identical rates of heat-related illness among underweight (%52) and obese (55%) admitted pilgrims in our cohort are striking and warrant mechanistic interpretation. In underweight individuals, reduced lean muscle mass limits sweat production, fluid reserves, and cardiovascular compensation under heat stress, while micronutrient depletion further impairs thermoregulatory capacity. In obese individuals, increased subcutaneous adipose insulation reduces conductive heat loss to the environment, and the higher metabolic cost of moving a larger body mass during the Hajj rituals elevates core temperature (21). The convergence of these distinct pathways at both BMI extremes produces comparable clinical vulnerability under the extreme ambient temperatures and sustained physical exertion characteristic of Hajj (21). From a public-health and operational standpoint, this finding supports targeted pre-travel risk stratification for pilgrims at both BMI extremes, dedicated hydration and active cooling resources at high-traffic ritual sites, and culturally adapted heat-illness prevention messaging.

Lastly, sociodemographic factors also influenced healthcare utilization. In univariate analysis, individuals from Southeast Asia, particularly those categorized as underweight and overweight, demonstrate a markedly lower probability of clinic visits compared to their European counterparts, who show a higher likelihood of attending clinics Similar observations have been made in studies of healthcare-seeking behavior, where cultural and socioeconomic differences, including health literacy and access to care, shape utilization patterns (22, 23). Furthermore, the data reveals that unemployed participants who were categorized as underweight also exhibit a reduced probability of clinic visits. This aligns with general health equity research showing that unemployed or lower-income individuals typically underutilize preventive and curative services due to reduced resources and health literacy (24). The multivariate analysis suggests that statistical significance is largely absent, except for the underweight and obese categories. It reveals that marital status is associated with a low probability of clinic visits, whereas employment status is correlated with a significant probability of such visits. Within the obese group, nationality does not show a statistically significant association with clinic visit probabilities.

Several **limitations** should be noted. First, the cross-sectional design limits causal inference between BMI and healthcare utilization (25). Second, the study did not assess physical activity which may confound the observed associations (26). Third, reliance on self-reported healthcare visits introduces potential recall and reporting biases (8). Finally, cultural and language differences among participants may have affected the accuracy of responses (4). Each limitation deserves further consideration. The cross-sectional design precludes any causal inference; the observed U-shaped pattern should be read as an association rather than a causal effect of BMI on utilization. Anthropometric data were self-reported, and self-reported height and weight are known to underestimate weight and overestimate height; the likely direction of resulting misclassification would tend to attenuate — rather than spuriously inflate — the BMI–utilization associations observed here. Healthcare visits were also self-reported, raising the possibility of recall bias that may differ by BMI category (for example, frequent visitors may recall encounters more accurately).

Several potentially relevant confounders were not collected in the questionnaire, including objective physical activity, hydration status, pre-existing comorbidities, and concurrent medication use, leaving room for residual confounding in the adjusted estimates. Individual length of stay in Makkah was not captured; although the data-collection window covered the full arrival-to-departure interval and most pilgrims travelled in standardized Hajj packages of broadly comparable duration (approximately one to three weeks), residual confounding by duration of exposure cannot be excluded, and we recommend that future studies record individual length of stay so utilization can be expressed as a per-pilgrim-day rate. Pilgrims who were severely ill or hospitalized and whose hospital visits were not documented in the data registry by foreign medical teams may be under-represented, which could under-estimate utilization at BMI extremes. Cultural and language differences may also have affected the accuracy of information documented in the data registry despite the involvement of multilingual foreign medical teams. The achieved sample size (*n* = 4,000) was slightly below the calculated minimum (*n* = 4,258), corresponding to a 1.55% realized margin of error rather than the planned 1.5%.Finally, the analysis modelled clinic visit as a binary outcome and assigned a single primary diagnosis per admitted participant; this approach does not capture the full burden of repeated or multi-diagnostic encounters, and future studies should model utilization as a count outcome (e.g., negative-binomial regression) to fully exploit information on repeated visits. The sample size of 4,000 pilgrims provided sufficient statistical power for these analyses, with greater than 80% power retained at *α*=0.05 for the observed effect sizes for the BMI  ×  clinic-visit association.

Overall, these findings indicate that BMI is associated with healthcare utilization during Hajj in a U-shaped manner, with both underweight and obese pilgrims showing higher clinic-visit and hospital-admission rates than the normal-weight reference group. The results emphasize the need for pre-travel risk stratification, targeted health education, and culturally competent healthcare planning that explicitly addresses the unique vulnerabilities of pilgrims at both BMI extremes during mass gatherings (4). Further prospective studies, especially those with longitudinal or interventional designs and individual-level length-of-stay data, are needed to better understand the mechanisms linking BMI to healthcare utilization during Hajj and to develop effective interventions (4).

## 5 Conclusions

The study reveals that Body Mass Index (BMI) is associated with healthcare utilization during the Hajj pilgrimage in a U-shaped manner: both underweight and obese pilgrims show higher rates of clinic visits and hospital admission than normal-weight pilgrims. Socioeconomic and regional factors also influence healthcare-seeking behaviors. Targeted public health strategies — including pre-travel medical assessments stratified by BMI, proactive management plans for pilgrims at both BMI extremes, are essential to minimize health burdens during mass gatherings.

## Data Availability

Data supporting this study are available from the corresponding author upon reasonable request.
